# Impact of COVID-19 lockdown on physical exercise among participants receiving the Promoting Activity, Independence and Stability in Early Dementia (PrAISED) intervention: a repeated measure study

**DOI:** 10.1186/s12877-022-03239-5

**Published:** 2022-07-21

**Authors:** Claudio Di Lorito, Veronika van der Wardt, Rebecca O’Brien, John Gladman, Tahir Masud, Rowan H. Harwood

**Affiliations:** 1 School of Medicine, University of Nottingham, Nottingham, NG7 2TU UK; 2 Philipps-University Marburg, Marburg, Germany; 3 Nottingham University Hospitals NHS Trust, Nottingham, UK; 4 School of Health Sciences, University of Nottingham, Nottingham, UK

**Keywords:** Sports Medicine, Telemedicine, PrAISED, Neurodegenerative disease, SARS-CoV-2

## Abstract

**Background:**

The potential decrease in daily physical activity associated with the COVID-19 pandemic lockdowns may have a negative impact on people living with dementia. Given the limited literature around the effects of home confinement in people living with dementia, this study investigated changes in physical exercise levels of participants in the intervention arm of the Promoting Activity, Independence and Stability in Early Dementia (PrAISED) Randomised Controlled Trial during the first COVID-19 national lockdown. It hypothesised that participants would maintain physical exercise levels.

**Methods:**

A repeated measure (three time points) study involving 30 participants (mean age = 78.0 years, 15 male and 15 female, 22 (73.0%) living with their primary caregiver), from four regions in England receiving the PrAISED intervention. PrAISED is an individually tailored intervention of physical exercises and functional activities. Trained therapists deliver therapy sessions over a period of 52 weeks. Study participants received therapy sessions via phone or video calling during the COVID-19 lockdown. This study investigated self-reported minutes of physical exercise recorded on study calendars for the months of February (i.e., baseline – pre-lockdown), May (i.e., T1 – during lockdown), and August (i.e., T2—post-lockdown) 2020.

**Results:**

Participants reported a statistically significant increase in activity levels between February and May (Wilcoxon Z = -2.013, *p* = 0.044) and a statistically significant decrease between May and August (Wilcoxon Z = -2.726, *p* = 0.004). No significant difference was found in the physical activity levels from pre- to post-lockdown (Wilcoxon Z = 0.485, *p* = 0.620).

**Conclusion:**

Despite concerns that the restrictions associated with the COVID-19 pandemic might lead to reductions in physical exercise, participants in receipt of the PrAISED intervention increased their amount of physical exercise during lockdown. Our findings support the potential of remote support for people living with dementia to help them maintain physical exercise levels in circumstances where face-to-face service provision is not possible.

**Trial registration:**

The PrAISED trial and process evaluation have received ethical approval number 18/YH/0059 from the Bradford/Leeds Ethics Committee.

The Clinical Trial Identifier for PrAISED is: ISRCTN15320670 (https://doi.org/10.1186/ISRCTN15320670). Registration was made on 04/09/2018.

**Supplementary Information:**

The online version contains supplementary material available at 10.1186/s12877-022-03239-5.

## Background

Dementia presents with a cluster of symptoms, including impairment in motor skills [1–4]. Research has found that being physically active generates numerous benefits in older adults living with dementia, including the maintenance of physical abilities, independence, and quality of life [5].

To achieve these benefits, the World Health Organisation (WHO) recommend at least 150 min of moderate-intensity aerobic physical activity or 75 or more minutes of vigorous-intensity aerobic physical activity per week, or an equivalent combination of the two [6]. Older adults who cannot exercise due to health conditions should engage in physical activity which is commensurate to their abilities [6]. The United Kingdom Chief Medical Officers' Physical Activity Guidelines argue that even minimal level of physical activity (e.g., standing) generates some health benefits, compared to being inactive [7].

The Promoting Activity, Independence and Stability in Early Dementia (PrAISED) Randomised controlled Trial (RCT) is evaluating the clinical and cost-effectiveness of an intervention promoting physical exercise in people living with dementia [8]. Participants in the intervention arm receive a programme of physical exercises that are designed to be at least of moderate intensity, based on to each participant’s individual ability, and functional activities (i.e., activities of daily living with an element of physical activity, such as going out for food shopping). It is delivered through up to 50 therapy sessions over a period of 52 weeks by trained physiotherapists, occupational therapists and rehabilitation support workers in the participants’ homes.

In March 2020, due to the rapid global spread of the Coronavirus disease 2019 (COVID-19) outbreak, the United Kingdom (UK) government mandated a national lockdown and social distancing measures, which required people to stay at home and allowed them to receive only strictly necessary in-home medical care, [9] making home visits from the PrAISED therapists impossible. Studies have found that lockdowns and social distancing measures impede individuals' mobility, daily activities, [10, 11] and social interactions [12]. As a result, an increase in the prevalence of psychological distress and disorder symptoms (e.g., depression, anxiety, negative feelings, emotional exhaustion, somatic symptoms, panic disorder) has been widely reported [10, 11]. To mitigate these risks and continue with the RCT, it was decided to deliver the PrAISED therapy sessions remotely using video calls and telephone. The PrAISED research team provided the therapists with plans and guidance on how to deliver PrAISED remotely (Additional file 1: Appendices 1, 2 and 3).

Studies on older adults have reported a decrease in physical activity during the COVID-19 lockdown [13]. Yet, there are currently few studies that investigate the effects of home confinement in people living with early dementia, and the potential associated decrease in daily physical activity levels. In a previous study published on BMC Geriatrics, [14] we reported preliminary qualitative evidence that, provided the support offered by the PrAISED team, some participants seemed to have been able to keep physically active during lockdown. The aim of this follow up study was to empirically investigate quantitative data around changes in the levels of physical exercise of participants who were in receipt of PrAISED through video/telephone during the first wave of the COVID-19 pandemic lockdown.

The research question was: Did the physical exercise levels of participants in receipt of PrAISED change throughout (i.e., before, during and after) the first wave of COVID-19 lockdown? Based on our previous preliminary study, [14] we hypothesised that through the remote support of the PrAISED team, the participants maintained their physical exercise levels throughout lockdown.

## Methods

This was a repeated measure study, using secondary data from the PrAISED RCT, [8] (but not reporting results from the RCT, which is ongoing). It made use of self-reported minutes of physical exercise compiled by all participants in the intervention arm of the PrAISED RCT on monthly calendars during the COVID-19 pandemic first lockdown in England.

The Clinical Trial Identifier for PrAISED is: ISRCTN15320670 (https://doi.org/10.1186/ISRCTN15320670). Registration was made on 04/09/2018.

### Sample

**Fig. 1:**
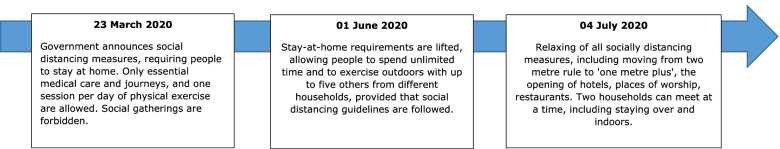
Timeline of the first national lockdown during the COVID-19 pandemic in England [16]

Inclusion criteria were:

- Aged 65 years or over
- Having a diagnosis of mild cognitive impairment or dementia
- Scoring 13–25 (out of 30) in the Montreal Cognitive Assessment (MoCA) [15]
- Being able to walk without human help, communicate in English, see andhear, perform neuropsychological tests, and give consent to participate
- Taking part in the PrAISED RCT in the intervention arm during the COVID-19 pandemic first wave national lockdown in England (Fig. 1)
- Agreeing to continue to take part in PrAISED when converted from in-home face-to-face to remote support using video calls and/or telephone.

### Setting

The participants were supported either through the telephone or video calling, based on their preference. For phone appointments, a suitable date and time for the therapy session was agreed between the therapist and the participant. On the agreed date, the therapist called the participant and held the session. If the participant needed support, the phone was put in “on speaker” mode, so that the caregiver (where present) could participate. For video calling appointment, the therapy sessions took place on Q Health (https://qhealth.io/), a National Health Service (NHS) Digital and NHS England-approved video patient consultation solution. Q Health allowed therapists and participants to set up and attend digital appointments. The system required access to technology (i.e., internet connection and a computer, tablet or smart phone) and the ability to download the Q Health application, to book the digital appointments and connect for the therapy session. The therapy team provided the participants with a set of instructions on how to operate the system and offered guidance via phone, if needed. Once installed, the participants were provided with a password to grant secure access, to select an appointment time and to start the video therapy session.

### Data collection

All participants signed informed consent prior to their inclusion in the trial. Demographic information about participants including age, gender, ethnicity, years of education, residency status, and location were collected at the time of involvement in the trial.

As part of the trial, the participants were asked to complete at home a diary of their PrAISED exercises in the form of a calendar (Additional file 2: Appendix 4). A previous study showed that return rates of 63–90% of these calendars are accurate reflections of activity levels in people living with dementia [17]. Instructions were provided to participants on how to fill in the calendar (Additional file 3: Appendix 5).

Minutes of PrAISED exercise were recorded by the participants each day, and at the end of each month, the calendar sheet (in paper format) listing all daily minutes of physical activity (i.e., one entry for each day of the month) was sent to the PrAISED team. Caregivers could support the participants to fill in the calendars, if needed. Using the participant ID on the calendar, the data were added to the trial database [8].

### Data analysis

Descriptive statistics and return rates were calculated. To investigate physical exercise levels of participants during lockdown, data from the following months were analysed:

- February 2020, used as proxy for pre-lockdown period in England – participants received face-to-face in-home visits only;
- May 2020, used as proxy for lockdown period in England – participants received remote support via phone/videoconferencing only;
- August 2020, used as proxy for post-lockdown period in England – participants received a mix of face-to-face in-home visits and remote support via phone/videoconferencing.

These months were selected based on government policy on social distancing in England [16].

To convert data to minutes of exercise per week, the daily minutes of exercise for each participant were added to obtain monthly figures. These were divided by the number of recordings in the month, and the result multiplied by seven. To test whether any differences in exercise levels were statistically significant across months, Wilcoxon signed-rank tests were conducted, comparing February and May, May and August, and August and February. Level of significance was set at *p* < 0.05. The analysis was completed on IBM SPSS [18].

### Patient and Public Involvement (PPI)

The PrAISED RCT includes two Patient and Public Involvement (PPI) contributors who have lived experience of caring for someone living with dementia. The PPI contributors were involved in the development of the RCT from its conception. Although they did not actively contribute to this manuscript, their feedback on the relevance of findings and implication for people living with dementia were sought and integrated in this study.

## Results

Thirty participants were included, with a mean age of 78.0 years (SD = 6.0; Range 66–88). Male and female participants were equally represented (*n* = 15; 50.0%). Most participants were white (*n* = 29; 97.0%) and lived with their primary caregiver (*n* = 22; 73.0%). The mean score at the MoCA [15] was 21.3 (SD = 3.3). Participants’ characteristics are reported in Table 1.

**Table 1:** Participants’ characteristics

		**n**	**%**	**Mean**	**Standard Deviation**	**Range**
**Age**				77.6	6.0	66—88
**Gender**	Male	15	50.0			
	Female	15	50.0			
**Ethnicity**	White	29	96.7			
	Black	1	3.3			
**Years of education**				13.1	3.9	8–25
**Residency status**	Living with caregiver	22	73.3			
	Living alone	7	26.7			
**Location**	Nottinghamshire	9	30.0			
	Derbyshire	10	33.3			
	Lincolnshire	7	23.3			
	Somerset	4	13.3			
**MoCA**^a^ **score**				21.3	3.3	13–25

^a^*MoCA* Montreal Cognitive Assessment [15]

One hundred and ninety-six therapy sessions were delivered to the participants in the three months, with an average of 2.2 sessions per month and a mean duration of 49.0 min per session (SD = 33.1) (Table 2). There was no significant difference in the duration of sessions between phone and video calls. The mean return rate of calendars was 76.0%, which is within range to reflect accurate activity levels [11].

**Table 2:** Information on sessions

Month	Sessions delivered (n)	Duration of session in minutes—mean (STD)	Frequency of sessions/month—mean	Mode of delivery – n (%)		
				Face-to-face delivery in participants’ homes	Remote delivery—phone	Remote delivery—videoconferencing
Feb 2020	84	76.0 (24.0)	2.8	84 (100.0)	-	-
May 2020	78	35.0 (20.0)	2.6	-	67 (86.0)	11 (14.0)
Aug 2020	34	36.0 (20.0)	1.1	1 (3.0)	25 (74.2)	8 (22.8)
Total	196	49.0 (33.1)	2.2	85 (-)	102 (-)	19 (-)

**Fig. 2:**
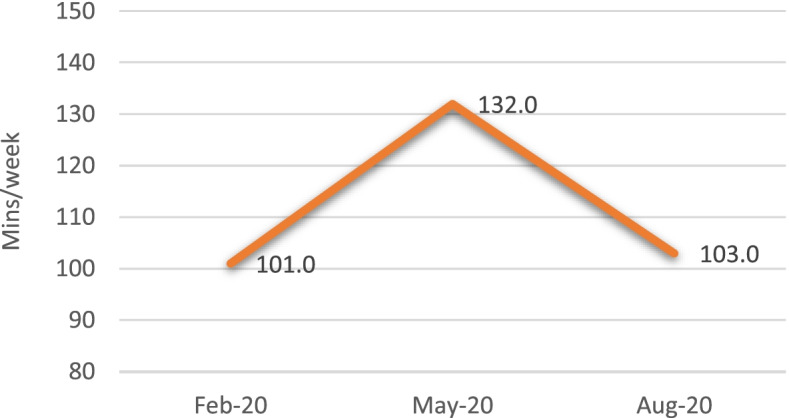
Average physical exercise time (minutes per week), self-reported by participants in the monthly calendars

In February 2020 (pre-lockdown), participants reported an average of 101.0 min per week of physical exercise; in May 2020 (during lockdown), they reported an average of 132.0 min per week; in August 2020 (post-lockdown), they reported an average of 103.0 min per week (Fig. 2). A Wilcoxon signed-rank test showed a statistically significant increase in activity levels between February 2020 and May 2020 (*Z* = -2.013, *p* = 0.040) and a statistically significant decrease in activity levels between May 2020 and August 2020 (*Z* = -2.726, *p* = 0.004). No statistically significant differences were found between February 2020 and August 2020 (*Z* = -0.485, *p* = 0.620).

## Discussion

This study found that before the lockdown, the participants undertook an average of 101.0 min per week of exercise (67% of the 150 min of weekly activity minutes recommended by the WHO [6]). Yet during the lockdown, participants increased the amount of physical exercise they undertook to an average of 132.0 min per week (88% of the WHO recommended amount). Once the lockdown restrictions were lifted, their levels of exercise returned close to pre-lockdown levels.

Our results are in contrast with existing research around physical activity levels during the COVID-19 pandemic, which seems to suggest that the overall population has experienced an increase in sedentariness [19–22]. A study with people with Mild Cognitive Impairment (MCI) also found a similar trend, with more than a third of one hundred and twenty-six participants reporting a reduction in physical activity and 70% reporting an increase in idle time [22]. The main difference with the previous studies is that our participants were consistently (i.e., up to one remote therapy session per week) supported throughout the lockdown.

In line with other programmes successfully increasing physical fitness in people living with dementia, [23, 24] and with findings from our companion paper, [14] we can speculate that PrAISED might have provided a resource that participants could make use of during lockdown to boost activity levels. The increase in activity levels might have been compounded by a lack of alternative activities (e.g., going out for walks) during lockdown, which may have made the participants more willing to “pass time” at home by doing something beneficial. In-home caregivers might also have spent more time at home during lockdown and might have been instrumental in encouraging this virtuous trend. In the absence of scientific literature comparing lockdown with post-lockdown activity levels, we can only speculate that with the return to “normality” post-lockdown, the participants reverted back to their pre-lockdown routines (and exercise trends).

Results from this study must be interpreted with caution, owing to a number of limitations. External validity is limited by the fact that the analysis focused on a small sample, a sub-set of the PrAISED study participants (i.e., those who were in the intervention group in PrAISED during the lockdown). Given the carefully designed nature of the PrAISED intervention, our results should not be assumed to be generalisable to all forms of activity support to people living with dementia, let alone to people without dementia. Additionally, because the PrAISED participants were supported during lockdown, findings about the increase in activity levels emerging from this study should not be generalized to people living with dementia who remained unsupported during lockdown. Another potential limitation pertains to the reliability of self-reported data on exercise levels in the calendars, which may have been affected by social desirability bias on the part of participants. However, it is assumed that, if occurring, social desirability bias would be consistent across the three timepoints, thus not impacting on changes of reported exercise level by the same participant. Finally, the PrAISED RCT has not ended, and so we are yet unable to determine the effect of increased activity, if any, upon health outcomes and well-being.

Nevertheless, results from this study present some important practical implications. The finding that the efficacy of the PrAISED intervention to increase activity was enhanced when the intervention delivery mechanism switched from face-to-face to remote during the lockdown raises the possibility that future service implementation could usefully include some amount of remote clinician-client contact. In the context of PrAISED, unfortunately, remote contact meant primarily telephone (84.3% of sessions were delivered through the phone in the lockdown months of May and August 2020), as most participants living with dementia experience a digital divide that made videoconferencing an option for the least cognitively deteriorated, most tech savvy and / or with a caregiver who can fully support them to navigate digital systems [25].

However, there is accumulating evidence on the benefits of health services via electronic information and telecommunication technologies in people living with dementia. Provided adequate infrastructure and support, digital interventions including exergaming, [26–31] computerised cognitive training, [32–34] assistive technology, [35] teleassistance, [36] and dyadic (i.e., person living with dementia and caregiver) care support, [37] have been linked to improvements in executive functioning, maintenance of physical abilities, reduction of frailty, delayed admission to care and service optimisation (reduction of staff travel time).

## Conclusion

Despite the limitations of this small, self-reported study, study finding suggest the need to further implementation of effective and inclusive individualised remote support for people living with dementia to help them increase their level of activity towards levels necessary to maintain their health in any circumstance where face-to-face support is unavailable. Investment in addressing the current challenges with telehealth should be prioritised, so that this model can be more widely implemented and successfully used in circumstances of social distancing or to ensure equitability of services with clients living with dementia who risk being underserved (e.g., rural areas).

## Supplementary Information

**Additional file 1.**

**Additional file 2.**

**Additional file 3.**

## Data Availability

The datasets generated and analysed during the current study are not publicly available to safeguard anonymity of participants, but are available from the corresponding author on reasonable request.

## Abbreviations

PrAISED: Promoting Activity, Independence and Stability in Early Dementia
SD: Standard Deviation
WHO: World Health Organisation
RCT: Randomised controlled Trial
MoCA: Montreal Cognitive Assessment
NHS: National Health Service
PPI: Patient and Public Involvement
MCI: Mild Cognitive Impairment
