# Supplementary material for: Impact of COVID-19 lockdown on physical exercise among participants receiving the Promoting Activity, Independence and Stability in Early Dementia (PrAISED) intervention: a repeated measure study
Source: BMC Geriatr. 2022 Jul 21;22:605. doi: 10.1186/s12877-022-03239-5 (PMC9299962; doi:10.1186/s12877-022-03239-5)
Supplement: Supplementary file 1 — Additional file 1. [file 12877_2022_3239_MOESM1_ESM.zip › Appendix 1.pdf]

Appendix 1. Plan for PrAISED2 Intervention in response to COVID19 restrictions (effective from 18.03.2020)

Immediate plan

The NIHR have stated that their funded studies should stop all non-essential face-to-face contact. The PrAISED intervention is not considered essential care and therefore we must stop all face-to-face contact with our participants.

However, because we have a duty of care to our patients considering many of them will be following the governments advice to reduce all social contact, we have devised a contingency plan to continue with the PrAISED intervention.

Intervention Group Participants

Therapy teams should contact all participants currently in the trial, or their carers if more appropriate, to explain the change in practice as below.

On-going Intervention Group Participants

Visits to participants should be replaced with **remote coaching** as per their normal schedule, in terms of frequency. For example, if you are seeing someone weekly, this should be continued until they reach the time to reduce to fortnightly. This is the example frequency schedule set out in the intervention manual, however, continue to adapt this as appropriate in the same way you have been doing.

- Month 1-2: bi-weekly
- Month 3-6: weekly
- Month 6-9: fortnightly
- Month 9-12: monthly

The length of the session may be much shorter depending on what is discussed.

The content of the session should be guided by the telephone coaching instructions below.

Some participants will not be suitable for remote sessions. If the participant is unable to engage with this type of coaching, the carer should be contacted to determine if they may be able to use the remote coaching to support the participant. If the remote coaching is of no benefit to either the participant or the carer, then a courtesy telephone call should be given each month to keep in touch with the carer or participant as appropriate.

Final sessions should be carried out via remote sessions as appropriate; these should be followed up with an end of therapy letter and any follow up material being provided using the post or email if appropriate.

New Intervention Group Participants

Intervention group participants seen by the research team but not yet seen by therapy team, or who are in the assessment phase of the intervention, should be informed that

they are not going to receive the PrAISED intervention until the current restrictions are lifted.

Control Group Participants

If you have completed the first control visit you can carry out up to two follow up visits by telephone as per the guidance below. If the first control visit has not yet been completed, please inform the participant that they are not going to receive the PrAISED intervention until the current restrictions are lifted.

Therapy Visit Log

Continue to complete the therapy visit log, via the hyperlink, for all remote sessions. Please put remote coaching in the comments box.

Medium-Term Plan

It is expected that PrAISED therapy staff at each site will deliver the immediate plan outlined above.

However, as the situation changes a medium-term plan (outlined below) may come into action.

If sites cannot deliver the remote coaching sessions due to therapy staffing difficulties, the university staff may have capacity to be able to support. The PI from each site must contact the University as soon as possible if this happens. For university staff to be able to do the telephone coaching sessions effectively, we will need to know:

- the participant's details (e.g., contact telephone number for them and the carer/informant)
- a synopsis of the previous intervention session and what they are currently working on

As each site is using different participant documentation systems, the PIs should liaise with Sarah Goldberg or Rebecca O'Brien, to form a contingency plan on how this will happen and how information is to be transferred and stored.

Telephone Coaching Instructions

Before making the telephone call make sure you have looked at NHS England current advice for the client group you are dealing with, as this is likely to change on a regular basis (https://www.nhs.uk/conditions/coronavirus-covid-19/). Participants may have concerns about their current situation that need answering before the participant will engage in coaching.

- Explain who you are and why you are calling.
- Ask how they are and discuss any immediate concerns (they may need signposting as appropriate).
- Review their current activity and exercise plan.
- Review what they are currently doing during their day.
- Be aware that for many participants all their activities may have stopped.

- Form a plan of what they can do within the **current** restrictions. For example, currently people are advised it is ok to walk outside as long as they stay 2m away from other people.
- Help them to make a daily plan of activities. For example, doing exercises more frequently, or if they are no longer walking outside can they walk in the garden or up and down the stairs to get some cardiovascular exercise.
- Advise against sitting for long periods of time. For example, use a timer to remind yourself to get up or get up during advert breaks in television programmes.
- If the person is able to and wants to, they could put you on speaker phone while you go through their exercise programme with them. Only do this if they have the capacity to do this with their telephone. This could also be done with their carer or family member or named informant.
- Be aware people may be feeling quite worried and/or low in mood. You may need to discuss the benefits of, and encourage them to continue to carry out daily activities or routines, such as getting dressed, or taking meals on time.
- Participants may raise safeguarding issues such as identifying they are low on medication and there is no one to help them with this. This will need to addressed using the usual safeguarding procedures.
- If participants are complaining of COVID 19 symptoms they should be encouraged to follow the current advice from NHS direct or to phone 111.

It is expected these telephone coaching guidelines will evolve as PrAISED therapists start conducting these sessions. Guidance can come from outside sources, e.g., RCOT have recently shared this online https://www.rcot.co.uk/staying-well-when-social-distancing. It is important that we share good practice and suggestions and will discuss these guidelines during our PrAISED Therapist Teleconferences.
