# Supplementary material for: Impact of COVID-19 lockdown on physical exercise among participants receiving the Promoting Activity, Independence and Stability in Early Dementia (PrAISED) intervention: a repeated measure study
Source: BMC Geriatr. 2022 Jul 21;22:605. doi: 10.1186/s12877-022-03239-5 (PMC9299962; doi:10.1186/s12877-022-03239-5)
Supplement: Supplementary file 1 — Additional file 1. [file 12877_2022_3239_MOESM1_ESM.zip › Appendix 2.pdf]

Appendix 2. First Session or Review Sessions - Telephone Coaching Checklist for physiotherapists and occupational therapists

1. If the participant has relevant information in their **participant file**, you may need to encourage them to locate this and have this with them at the beginning of the session
2. Complete the **therapy visit log**, via the hyperlink, for all telephone calls. Please update goals if relevant.
3. Continue to complete the **Frequency and Intensity Decision Support Tools** and email a copy to Vicky/Louise if you are changing the frequency of sessions.

**Telephone Coaching - Praised Intervention**	**Comments**
**Goals**	
Review current **activity levels** and **PrAISED goals** and agree which goals can continue.<br><br>**Form a plan** on what participant can do within the current restrictions e.g. if a walking goal, they are able to walk outside once a day staying 2 metres away from others (non - family), walk in the garden, use the stairs.<br><br>Put unachievable goals impacted on by COVID restrictions 'on hold'.	
**Identify activities they enjoy**	
Think about which regular activities are most important to participant; ones that they are doing during the day. What are the important elements to these?<br><br>Can you **adapt** them for the PrAISED programme to carry out in the home? For example, instead of a class, following an online strength and balance routine.<br><br>Think about whether the participant's needs to feel **competent** and **autonomous** are being met.<br><br>Are they **using all the space available to them**, ie. Garden or hobby in spare room?	
**Routines**	
Routines provide structure and purpose.<br><br>Establish **a daily routine** with the participant and set daily goals to provide purpose and a sense of achievement. This might include working through that list of the things they have been meaning to do but never get round to.	

Can you help them build activities or exercise into **habits** which will help them continue longer term?<br><br>Establish a balance of a **weekly routine** so they have a good mix of work (activities that have to be done), rest and leisure.<br><br>Provide them with a weekly plan if appropriate.	
**Exercise Programme**	
Are they able to continue with the exercise programme you have previously provided?<br><br>Do they need to **identify support** with this either a member of the household or a family member that can do via technology?<br><br>Are they able to continue with **balance, strength** and **dual tasking activities**?<br><br>If not can you encourage them to **do activities of daily living** that cover these three areas?	
**Tapering/Long Term Engagement**	
If the participant is coming to the end of the 12 month intervention period. Discuss how they can -<br>• independently continue to work on their goals<br>• Remain as active as possible<br>• Identify sources for further support<br>• Explore other resources they may be able to access	

Staying Well and Social Distancing	Comments
**Regular Activity**	
Encourage participant to **avoid staying still for too long.** Exercise and regular movement will maintain fitness and strength. Use a timer to remind themselves not to sit for too long.<br><br>Encourage activity up to **150 minutes a week**	
**Relatedness**	
Encourage participant to **keep in touch** with family, friends and neighbours to help them understand how they feel and how they can help.<br><br>Suggest they arrange to speak to someone most days on the phone, through social media or over the garden fence.<br><br>Age UK and Silverline have people to speak to.	
**Self-care**	
**If someone feels worried or low in mood** – try and identify the triggers that make them feel low and look for ways to reduce or manage them.<br><br>Encourage participant to **take care of themselves**. Eat and drink healthily with plenty of fruit, vegetables and water, to help boost immune system and energy levels.	
**Sleep**	
Encourage participant to have **a good sleep routine**. If they are struggling, try avoiding tea and coffee in the late afternoon and evening, take a bath, using blackout curtains, listening to gentle music or deep breathing exercises.	

Safeguarding	
If a Safeguarding issue is raised e.g. participant without meds or food:<br><br>- contact informant in first instance,<br><br>- if unresolved it should go to Safeguarding trust policies and contact local social care need if appropriate.	
**COVID -9 symptoms**	
If participant is complaining about COVID-19 symptoms encourage them to follow current advice from NHS direct or to phone 111	
**Provide Information**	
Send details of resources they can use at home – see resource sheet<br><br>Issue RCOT 'top tips' sheet on staying well when social distancing<br><br>https://www.rcot.co.uk/staying-well-when-social-distancing	
**Technology**	
Check technology available to use at home that you may be able utilise in the future and if there is anybody who can support them in using this	

There are also additional **Resources for participants and therapists on PrAISED for during the coronavirus isolation restrictions**
