# Supplementary material for: Impact of COVID-19 lockdown on physical exercise among participants receiving the Promoting Activity, Independence and Stability in Early Dementia (PrAISED) intervention: a repeated measure study
Source: BMC Geriatr. 2022 Jul 21;22:605. doi: 10.1186/s12877-022-03239-5 (PMC9299962; doi:10.1186/s12877-022-03239-5)
Supplement: Supplementary file 1 — Additional file 1. [file 12877_2022_3239_MOESM1_ESM.zip › Appendix 3.pdf]

Appendix 3. Review Sessions - Telephone Coaching Checklist for rehabilitation support workers

Telephone Coaching Checklist – Praised Intervention

1. Complete the **therapy visit log**, via the hyperlink, for all telephone calls. Please update goals if relevant.
2. Continue to complete the **Frequency and Intensity Decision Support Tools** and email a copy to Vicky/Louise if you are changing the frequency of sessions.
3. Put a number in the RSW column on the **Visit and Task Tracker on TEAMS** - change the colour of the font or background to identify that it was a telephone visit (the ratio of therapist/RSW is now variable so we will record all telephone sessions as RSW sessions).

**Telephone Coaching - Praised Intervention**	**Comments**
Ask how the participant is and talk through any immediate concerns<br><br>**Discuss** which regular activities are most important to participant; ones that they are doing during the day.<br><br>What are the important elements to these?<br><br>Can you **adapt** them for the PrAISED programme to carry out in the home? For example, instead of a class, following an online strength and balance routine.<br><br>Can they do **ADL activities** that challenge balance, promote strength or include dual tasking?	

**Staying Well and Social Distancing**	**Comments**
**If someone feels worried or low in mood** – try and identify the triggers that make them feel low and look for ways to reduce or manage them.	
Encourage a **daily routine** with the participant and set daily goals to provide purpose and a sense of achievement. This might include working through that list of the things they have been meaning to do but never get round to.<br><br>Can you help them build activities or exercise into **habits** which will help them continue longer term?	

Encourage balance in a **<u>weekly</u> routine** so they have a good mix of work (activities that have to be done), rest and leisure.	
Encourage participant to **keep in touch** with family, friends and neighbours to help them understand how they feel and how they can help.<br><br>Suggest they arrange to speak to someone most days on the phone, through social media or over the garden fence.<br><br>Age UK and Silverline have people to speak to.	
Encourage participant to **take care of themselves**.<br><br>Eat and drink healthily with plenty of fruit, vegetables and water, to help boost immune system and energy levels.	
Encourage participant **to avoid staying still for too long.** Exercise and regular movement will maintain fitness and strength.<br><br>They could use a timer to remind themselves not to sit for too long, or get up and walk around in ad breaks etc	
Encourage participant to have a **good sleep routine**. If they are struggling, try avoiding tea and coffee in the late afternoon and evening, take a bath, using blackout curtains, listening to gentle music or deep breathing exercises.	
Refer to RCOT 'top tips' sheet on staying well when social distancing if needed<br><br>https://www.rcot.co.uk/staying-well-when-social-distancing	

If a **safeguarding** issue is raised e.g. participant without meds or food:<br><br>- contact informant<br><br>- if unresolved the case should be further discussed with local clinical services and the Oxford Praised study team	
If participant is complaining about **COVID-19 symptoms** encourage them to follow current advice from NHS direct or to phone 111<br><br>https://www.nhs.uk/conditions/coronavirus-covid-19/	

There are also additional **Resources for participants and therapists on PrAISED for during the coronavirus isolation restrictions** on TEAMS
