# Supplementary material for: Impact of COVID-19 lockdown on physical exercise among participants receiving the Promoting Activity, Independence and Stability in Early Dementia (PrAISED) intervention: a repeated measure study
Source: BMC Geriatr. 2022 Jul 21;22:605. doi: 10.1186/s12877-022-03239-5 (PMC9299962; doi:10.1186/s12877-022-03239-5)
Supplement: Supplementary file 2 — Additional file 2. [file 12877_2022_3239_MOESM2_ESM.pdf]

Appendix 4. Calendar sheet

PrAISED2 Study Calendar - <u>please complete every day</u>

P r A I S E D
Promoting Activity, Independence and Stability in Early Dementia and Mild Cognitive Impairment

August 2020

Participant ID: _ _ _ _ Patient Participant Initials: ______

Day	Falls		PrAISED Exercises	
	Number of falls	If you had a fall, what happened?	Yes / No	Time in minutes
example	*0*	*n/a*	*No*	*n/a*
1, Sunday				
2, Monday				
3, Tuesday				
4,Wednesday				
5, Thursday				
6, Friday				
7, Saturday				
8, Sunday				
9,Monday				
10,Tuesday				
11,Wednesday				
12,Thursday				
13,Friday				
14,Saturday				
15,Sunday				
16,Monday				
17,Tuesday				
18,Wednesday				
19,Thursday				
20,Friday				
21,Saturday				
22,Sunday				
23,Monday				
24,Tuesday				
25,Wednesday				
26,Thursday				
27,Friday				
28,Saturday				
29,Sunday				
30,Monday				
31,Tuesday				

Monthly Service Use Questionnaire:

Please complete the short questionnaire below at the end of the month.

In the last month, have you had any A&E / Emergency Department attendances?

☐ Yes ☐ No

If yes, how many? ☐

If yes, please tell us what happened in **Box 1**.

In the last month, have you had any paid help *(e.g. home carer, cleaner, sitting service)?*

☐ Yes ☐ No

If yes, how many hours? ☐

In the last month, have you had any unpaid help (informal care from family and friends)?

☐ Yes ☐ No

If yes, how many hours? ☐

In the last month, have you had any nights in a care home / respite care?

☐ Yes ☐ No

If yes, how many nights? ☐

In the last month, have you had contact with a nurse of any kind?

☐ Yes ☐ No

If yes, how many contacts? ☐

In the last month, have you had contact with a GP?

☐ Yes ☐ No

If yes, how many contacts? ☐

If yes, please tell us what happened in **Box 1**.

Box 1. Injuries or symptoms during or following your exercises or activities

Date	What happened?	Date	What happened

If you need more space, please use the back of this sheet

<u>Please detach and return in the pre-paid envelope provided</u>
