# Supplementary material for: Impact of COVID-19 lockdown on physical exercise among participants receiving the Promoting Activity, Independence and Stability in Early Dementia (PrAISED) intervention: a repeated measure study
Source: BMC Geriatr. 2022 Jul 21;22:605. doi: 10.1186/s12877-022-03239-5 (PMC9299962; doi:10.1186/s12877-022-03239-5)
Supplement: Supplementary file 3 — Additional file 3. [file 12877_2022_3239_MOESM3_ESM.pdf]

Appendix 5. Instruction for participants

University of Nottingham UK | CHINA | MALAYSIA

Nottingham University Hospitals NHS Trust

PrAISED Promoting Activity, Independence and Stability in Early Dementia and Mild Cognitive Impairment

Calendar Guide

Falls

- Tell us if you have had a fall, by writing the number of falls each day
- If you had a fall, briefly describe what happened.
- If you did not have a fall, please write '0'. Please do not leave it blank.

PrAISED Exercises

If you have been allocated to Therapy Package 2, please complete this column:

- Tell us if you have done any of your PrAISED exercises, by writing Yes or No for each day.
- If you have done your exercises, please tell us how many minutes you did these for on that day.
- You can include the time you have done the exercises when the therapist visits you.

Monthly Service Use Questionnaire

Answer these questions when you reach the end of the month for each calendar page (i.e. each month).

- Tick yes or no for each question.
- If yes, tell us the number of times / hours (as appropriate) you have used the service. You can use the back of the page to write notes or further explanations if you wish.
- If you have had any A&E / Emergency Department attendances in the last month, please tell us what happened in the 'Injuries or Symptoms' box (**Box 1**.).

Injuries or Symptoms

If you hurt yourself or experience any unpleasant symptoms due to any exercises or activities:

- Tell us what date this happened
- Describe what happened. For example: 'felt dizzy whilst doing sitting to standing exercises'.

Once you have reached the end of the month:

- Tear off the calendar page and post it to us in a prepaid envelope provided.
  Your envelopes will be sent out one at a time towards the end of the month.

If you have any questions about the calendars, please contact:
